# Mentalizing, Resilience, and Mental Health Status among Healthcare Workers during the COVID-19 Pandemic: A Cross-Sectional Study

**DOI:** 10.3390/ijerph20085594

**Published:** 2023-04-20

**Authors:** Teodora Safiye, Medo Gutić, Jakša Dubljanin, Tamara M. Stojanović, Draško Dubljanin, Andreja Kovačević, Milena Zlatanović, Denis H. Demirović, Nemanja Nenezić, Ardea Milidrag

**Affiliations:** 1 Faculty of Medical Sciences, University of Kragujevac, Svetozara Markovića 69, 34000 Kragujevac, Serbia; 2 Public Health Institution Health Center “Dr Branko Zogovic”, Hridska bb, 84325 Plav, Montenegro; 3 Faculty of Medicine, University of Belgrade, Dr Subotića 8, 11000 Belgrade, Serbia; 4 Department of Cardiology, University Clinical Hospital Center Zemun, Vukova 9, 11080 Belgrade, Serbia; 5 Faculty of Philology and Arts, University of Kragujevac, Jovana Cvijića bb, 34000 Kragujevac, Serbia; 6 Department of Pulmonology, University Clinical Hospital Center Zvezdara, Dimitrija Tucovića 161, 11120 Belgrade, Serbia; 7 Department of Cardiopulmonary Rehabilitation, Institute for Rehabilitation Belgrade, Sokobanjska 17, 11000 Belgrade, Serbia; 8 Department of Medical Studies Ćuprija, Academy of Educational and Medical Vocational Studies Kruševac, Bulevar Vojske bb, 35230 Ćuprija, Serbia; 9 Department of Psychology, Faculty of Philoshopy and Arts, State University of Novi Pazar, Vuka Karadžića 9, 36300 Novi Pazar, Serbia

**Keywords:** COVID-19, mental health status, mentalizing, depression, anxiety, stress, resilience, Serbia, doctors, nurses

## Abstract

The COVID-19 pandemic has caused unprecedented stress on healthcare professionals worldwide. Since resilience and mentalizing capacity play very important preventive roles when it comes to mental health, the main goal of this study was to determine whether the capacity for mentalizing and resilience could explain the levels of depression, anxiety, and stress among healthcare workers during the COVID-19 pandemic. The study was conducted in Serbia on a sample of 406 healthcare workers (141 doctors and 265 nurses) aged 19 to 65 (M = 40.11, SD = 9.41). The participants’ mental health status was evaluated using the Depression, Anxiety, and Stress Scale—DASS-42. The Reflective Functioning Questionnaire was used to evaluate the capacity for mentalizing. Resilience was assessed using the Brief Resilience Scale. The results of the correlation analysis showed that there were negative correlations between resilience and all three dimensions of mental health status: depression, anxiety, and stress. Hypermentalizing was negatively correlated with depression, anxiety, and stress, while hypomentalizing was positively correlated. Hierarchical linear regression analysis showed that both resilience and hypermentalizing were significant negative predictors of depression, anxiety, and stress, and that hypomentalizing was a significant positive predictor of depression, anxiety, and stress. Furthermore, socioeconomic status was a significant negative predictor of depression, anxiety, and stress. Marital status, number of children, and work environment were not statistically significant predictors of any of the three dimensions of mental health status among the healthcare workers in this study. There is an urgent need to establish and implement strategies to foster resilience and enhance the capacity for mentalizing among healthcare workers in order to minimize the devastating effects of the COVID-19 pandemic on mental health.

## 1. Introduction

Severe acute respiratory syndrome coronavirus 2, also referred to as SARS-CoV-2, is a highly contagious, morbific virus that belongs to the group of coronaviruses. With its outbreak in China in 2019, there was a pandemic of an acute respiratory disease called ‘coronavirus disease 2019’ (COVID-19), which resulted in endangering the health of the population, as well as public safety. The World Health Organization announced a pandemic in March 2020 due to the involvement of over 110 countries and territories around the world [1]. In addition to the fact that COVID-19 caused changes in daily lifestyle, it had devastating effects on both the physical and mental health of individuals [2,3]; a special impact was noted in the field of mental health of healthcare workers (HCWs) worldwide [4]. The consequences of the COVID-19 pandemic on mental health are most often manifested in the form of increased symptoms of stress, anxiety, frustration, and depression. Common psychological reactions associated with changing lifestyles during a pandemic are generalized fear and anxiety, which are usually caused by information about the easy transmissibility of the virus and rapid escalation of new cases of infection, as well as previous information from the media that cause restlessness and a sense of uncertainty [3]. The absence of social interaction due to isolation measures additionally contributes to the experience of stress since social activities and support help regulate emotions, overcome stress, and maintain resilience when facing difficult life events [2].

An increased level of distress is associated with working in healthcare, even under regular circumstances, and in this context, anxiety, depression, sleep disorders, and burnout syndrome among healthcare workers were described [5,6]. However, throughout the course of fighting against the pandemic, healthcare workers were at risk of facing enormous amounts of stress, as a large number of medical personnel became infected due to the outbreak of SARS-CoV-2, which further increased the psychological stress of their colleagues [1]. In addition, they faced not only a greater risk of infection and the fear of infection and the spread of the virus to their loved ones but also emotional disorders, sleep problems, isolation, lack of contact with their families, extended shifts, and physical exhaustion [4,7]. Healthcare workers are responsible for other people’s lives; therefore, all these psychological burdens, especially among medical staff on the frontline, can lead to a strong negative impact on their mental health [8]. There is increasing evidence to suggest that COVID-19 may be an independent risk factor for stress in healthcare workers. In a review of six studies, Spoorthy et al. reported that healthcare workers experienced significant levels of stress, anxiety, depression, and insomnia due to the COVID-19 pandemic [9]. It was also shown that in addition to psychological variables, such as poor social support and low self-efficacy, certain sociodemographic variables, such as gender, profession, age, and place of work negatively affected mental health, more precisely, they were associated with increased symptoms of stress, anxiety, and depression among healthcare workers [9]. It is considered that resilience [6,10] and good mentalizing capacity [11] play very important roles in reducing these mental health symptoms and in preserving mental health while facing crisis situations, such as the pandemic.

When we talk about psychological resilience, we are talking about the ability of people to more easily tolerate increased levels of stress and to function adequately in difficult circumstances. In the literature, it is most often stated that resilience is a general personality trait that implies the capacity for effective adaptation and the capacity to sustain or regenerate mental health under challenging circumstances [12]. Smith et al. believed that resilience is not only a process of manifesting positive perspectives and coping mechanisms but also the individual’s capacity to quickly and efficiently recover from stressful experiences [13]. Multidisciplinarity in the approach to this problem also led to the evolution and change of definitions known so far, along with the evolution of the scientific understanding and knowledge of this issue. Thus, over time, several sources of resilience that are interconnected were identified: (1) Individual characteristics, such as openness, extraversion, agreeableness, and cooperation; internal locus of control; optimism; cognitive flexibility; emotional regulation; hope; resourcefulness; and adaptability. (2) Biological factors—an unfavorable early environment can affect the size, neuronal networks, receptor sensitivity, and the synthesis and uptake of neurotransmitters in the developing brain. These structural alterations in the brain can significantly increase or decrease susceptibility to psychopathology in the future, as well as the capacity to control negative emotions and, consequently, resilience to adversity. These changes can lead to the impairment of a person’s ability to cope with emotions. (3) Environmental and systemic factors—social support from family and peers, secure bond with the mother, a stable family, an appropriate relationship with a non-abusive parent, effective parenting techniques, etc. [14]. Well-developed resilience was cited even before the COVID-19 pandemic as a trait that could enable healthcare workers to recover more easily from various difficulties and could be acquired through an appropriate training program [6].

Recently, the capacity for mentalizing was cited as an important characteristic of resilience, which implies the human ability to understand one’s own and others’ intentional mental states, which affects the general resilience of the personality, i.e., the ability to successfully adapt to challenges and stress. This aspect of reflective personality functioning can also be characterized as a person’s ability to understand himself/herself and others, both in terms of subjective mental states and in terms of mental processes. In essence, the concept of mentalizing refers to how an individual explains his behavior and the behavior of others through the interpretation of the reasons, causes, and motives of events [15]. When we talk about the process of mentalizing itself, we are talking about the interdependence between subjective understanding of the mental states of ourselves and others, which affects our behavior and allows us to feel in control of our thinking and the way we act, as well as the way we take in, analyze and interpret social information from the environment around us [16]. However, as stated by some authors [17], in the process of mentalizing, we can distinguish between two qualitatively different, wrong ways of interpreting signals from the environment. These are hypomentalizing and hypermentalizing. Making too many assumptions about intentional mental states, without thinking critically about their truth, is called hypermentalizing. It manifests as excessive confidence in the veracity of one’s own views about the nature of the mental processes that underlie one’s actions and arises as a result of the individual’s incorrect beliefs that other persons have similar or the same intentional mental states. In contrast, hypomentalizing refers to the lack of assumptions about the mental states that determine behavior in interpersonal interaction, which manifests itself as uncertainty regarding the accurate assessment of the mental states underlying some behavior [17]. Bearing in mind the mentioned characteristics of mentalizing, it is crucial to implement preventive or early interventions that encourage the ability to mentalize, which contributes to increasing resilience to life stressors, and thus, protecting individuals from the influence of disturbing factors and preserving their mental health [18]. This is especially important nowadays when every individual and society is faced with numerous stressful situations, such as the COVID-19 pandemic and its consequences.

With all of this in mind, the primary objective of this study was to determine whether mentalizing capacity and resilience could account for the levels of stress, anxiety, and depression experienced by healthcare professionals during the COVID-19 epidemic in Serbia. We hypothesized that significant relationships would be found between resilience, as well as impairments in mentalizing capacity, and the mental health status of HCWs (operationalized through levels of depression, anxiety, and stress). As far as we know, this study is one of the few that examined the specificity of the protective impact of mentalizing capacity on mental health in a sample of healthcare workers facing the COVID-19 pandemic.

## 2. Materials and Methods

### 2.1. Study Design, Participants, and Procedures

A cross-sectional study design was adopted. The current study is part of a large, self-funded project entitled “Burnout syndrome and indicators of mental health of workers during the COVID-19 pandemic in Serbia”, led by Teodora Safiye. The Institutional Review Board of the University of Belgrade’s Faculty of Philosophy, Department of Psychology, approved the project (approval number: #2021-58). The protocols used in the present study adhered to the Declaration of Helsinki’s guidelines for medical research with human individuals [19]. Prior to the study’s recruitment, each subject provided a written statement of informed consent. Each participant was given comprehensive information on their rights to inquire about their involvement in the study and to withdraw at any time without consequence. The study report did not include the names of the participants, and all data gathered from them was protected so that just the investigators could access it. The Raosoft Sample Size Calculator (available online: http://www.raosoft.com/samplesize.html (accessed on 1 May 2021)) was used to determine the necessary sample size. A sample of 377 respondents was estimated using the following assumptions: a 5% margin of error, a 95% confidence interval, and a population size of 20,000. Doctors and nurses from Serbia who were living in Serbia, actively caring for patients at the University Clinical Center Kragujevac at the time of the study, and between the ages of 18 and 65 were eligible to participate in the study. After the approval of the Ethics Committee of the University Clinical Center Kragujevac, we started gathering data using the paper-and-pencil survey method, in which survey participants had to complete a paper-based survey by hand. The research’s aims were disclosed to possible participants in Serbian from the very beginning of the application of the questionnaire, and participation was voluntary, free of charge, and with informed consent. At the same time, the respondents were guaranteed the confidentiality and anonymity of the data obtained. The research was carried out between 15 July 2021 and 5 February 2022 at the University Clinical Center Kragujevac in Serbia during the height of the COVID-19 outbreak in Serbia [20].

### 2.2. Measures

The Depression, Anxiety, and Stress Scale—DASS-42 [21] was used to evaluate the participants’ mental health status, namely, to measure depression, anxiety, and stress. The DASS-42 scale consists of 42 items and includes three subscales: depression, anxiety, and stress. According to the authors, the items of the depression subscale refer to experiences of dysphoria (e.g., “I felt sad and miserable”), anhedonia (e.g., “I couldn’t seem to experience any positive feelings at all”), hopelessness (e.g., “I felt that I had nothing to look forward to”), devaluing oneself (e.g., “I felt I wasn’t worth much as a person”), and devaluing life (e.g., “I felt that life wasn’t worthwhile”); the anxiety subscale refers to experiences of apprehension, high tension, and helplessness (e.g., “I felt I was close to panic”); and the stress subscale refers to high arousal of the organism and negative emotions arising as a result of unpleasant or threatening events (e.g., “I found it difficult to relax”). The score on the depression subscale is calculated by adding the points from questions 3, 5, 10, 13, 16, 17, 21, 24, 26, 31, 34, 37, 38, and 42. The score on the anxiety subscale is calculated by adding the points from questions 2, 4, 7, 9, 15, 19, 20, 23, 25, 28, 30, 36, 40, and 41. The score on the stress subscale is calculated by adding the points from questions 1, 6, 8, 11, 12, 14, 18, 22, 27, 29, 32, 33, 35, and 39. The total score on each of the three mentioned subscales can range from 0 to 42. Respondents used a 4-point Likert-type scale, from 0—not at all to 3—mostly or almost always, to assess their degree of agreement without dwelling too much on the items. Since this research examined depression, anxiety, and stress as indicators of the respondents’ mental health status (not as personality traits), the respondents were instructed to carefully read each item and mark the answer that best described how they felt in the previous 7 days [21]. The original long form of the DASS-42 questionnaire has had very good reliability, where the Cronbach alpha coefficient was above 0.8 in previous studies [22,23].

Resilience was measured using the Brief Resilience Scale (BRS), which was developed by Smith et al. [13]. This scale assesses the construct of resilience, which is understood as an individual’s ability to cope with obstacles from the environment and recover from stressful circumstances. The short resilience scale has very good reliability, where the Cronbach alpha coefficient was above 0.8 in previous research [6,13]. Six items make up the one-dimensional, brief resilience scale. Resilience is endorsed by three items (e.g., “I typically come through challenging times with little trouble”), while it is dismissed by three items with reverse scoring (e.g., “I have a hard time making it through stressful events”). The mean of all six items represents the final score on this scale. Respondents had the option of selecting one response on a five-point Likert-type scale, from 1—completely false to 5—completely true [13]. A shortened version of the Reflective Functioning Questionnaire (RFQ-8) [17], which is advised for research to prevent subject fatigue, was used to assess mentalizing capacity. The RFQ-8 has two subscales: (1) the subscale of certainty in one’s own assessment of mental states (RFQ-c), where high scores denote the phenomenon of hypermentalizing, and (2) the subscale of uncertainty in one’s own ability to assess one’s own and others’ mental states (RFQ-u), which denotes the phenomenon of hypomentalizing. The RFQ-c subscale measures how strongly a person disagrees with statements like “People’s thoughts are a mystery to me” in order to determine how confident a person is in their ability to effectively evaluate both their own and others’ mental states. The RFQ subscale assesses a person’s level of insecurity regarding their capacity to judge both their own and other people’s mental conditions. The degree to which a person agrees with phrases like “I don’t always know why I do what I do” is used to measure it. Low values indicate optimal mentalizing, while high scores indicate hypomentalizing [24,25]. Responses are rated on a seven-point Likert scale, with 1 representing “I do not agree at all” and 7 representing “I completely agree”. The responses received from respondents on both RFQ-8 subscales could range from 0 to 3. In earlier studies, the RFQ demonstrated high reliability; the Cronbach alpha coefficient was 0.70 or higher [24,25]. A special questionnaire was developed especially for this project in order to evaluate sociodemographic, work, and COVID-19-related characteristics. Based on previous studies that dealt with examining the mental health status of healthcare workers [26,27,28,29], factors such as gender (male = 1, female = 2), age, profession (doctors = 1, nurses = 2), work environment during the COVID-19 pandemic (frontline healthcare workers = 1, non-frontline healthcare workers = 2), socioeconomic status (ranging from 1 = very poor to 5 = excellent), marital status (married = 1, single = 2), and number of children (no children = 1, one child = 2, two or more children = 3) were used as the study’s control variables.

### 2.3. Statistical Analysis

Inadequate answers, responses from participants who did not meet all the criteria needed for inclusion in the study, and responses from respondents who did not answer all the questionnaire questions were all excluded based on direct inspection prior to the statistical analysis of the data gathered. SPSS Statistics software (IBM SPSS Statistics for Windows, Version 22.0, Armonk, NY, USA) was used for statistical processing and data analysis. Measures of descriptive statistics included number (N), frequency (%), mean values, standard deviations (SD), minimum, maximum, skewness, and kurtosis. As an indicator of internal consistency, Cronbach’s alpha coefficient was used to evaluate the scales’ reliability. Categorical variables were transformed into numerical values for statistical purposes. Pearson’s correlation coefficients, along with significance tests, were utilized to describe the relationship between the studied variables. A multiple hierarchical regression analysis was utilized to assess whether the resilience and mentalizing aspects were significant predictors of depression, anxiety, and stress as dependent variables separately.

## 3. Results

### 3.1. Characteristics of the Analyzed Sample

The sociodemographic and work-related characteristics of the respondents are shown in Table 1. Of the tested healthcare workers, the majority were females (65.8%), nurses (65.3%), and married individuals (71.7%). Of the 406 healthcare workers who participated in the study, 203 were frontline healthcare workers (64 doctors and 139 nurses), while 203 were non-frontline healthcare workers (77 doctors and 126 nurses). The doctors and nurses in this study were between the ages of 19 and 61 and 26 and 62, respectively. On a scale of 1 to 5, the majority of respondents (56.4%) gave their socioeconomic situation a score of 3, which is considered to be good.

### 3.2. Measures of Descriptive Statistics of the Mentalizing, Resilience, and DASS-42 Dimensions

Table 2 shows the descriptive statistics measures and the reliability of the used scales. The results on these scales’ score distribution did not differ significantly from the normal distribution, according to the values of skewness and kurtosis, which varied from −1 to 1. When depression and hypomentalizing were measured, there was only a minor deviation, which indicated that the majority of respondents had depression and hypomentalizing scores that were below average. It was expected that all of the instruments used in this study would have satisfactory, good, or excellent reliability as determined by Cronbach’s alpha coefficient.

### 3.3. Correlations between the Investigated Variables

Table 3 shows the correlations between the variables. Our findings indicated that with a higher degree of resilience of the healthcare workers, their depression, anxiety, and stress were lower (r = −0.51, *p* < 0.01; r = −0.51, *p* < 0.05; r = −0.53, *p* < 0.01, respectively). With a greater degree of certainty in one’s ability to assess intentional mental states, i.e., hypermentalizing, the experience of depression, anxiety, and stress decreased (r = −0.42, *p* < 0.01; r = −0.43, *p* < 0.01; r = −0.49, *p* < 0.01, respectively). With a higher level of hypomentalizing, the degree of depression, anxiety, and stress increased (r = 0.40, *p* < 0.01; r = 0.46, *p* < 0.01; r = 0.53, *p* < 0.01, respectively).

Previous research findings showed that some control variables were important for mental health status [26,27,28,29]. Our findings suggested that females had more depression, anxiety, and stress (r = 0.13, *p* < 0.01; r = 0.25, *p* < 0.01; r = 0.17, *p* < 0.01, respectively), and at the same time, lower resilience (r = −0.18, *p* < 0.01). With the increase in the age of the respondents (r = −0.14, *p* < 0.01) and the number of children they had (r = −0.11, *p* < 0.05), the level of stress decreased. With the experience of higher socioeconomic status, respondents experienced less depression, anxiety, and stress (r = −0.28, *p* < 0.01; r = 0.20, *p* < 0.01; r = −0.19, *p* < 0.01, respectively). The findings suggested that nurses had higher levels of depression, anxiety, and stress (r = 0.10, *p* < 0.05; r = 0.20, *p* < 0.01; r = 0.10, *p* < 0.05, respectively) than doctors. Frontline healthcare workers were found to have higher levels of depression, anxiety, and stress (r = −0.10, *p* < 0.05; r = 0.10, *p* < 0.05; r = 0.13, *p* < 0.01, respectively) than non-frontline healthcare workers.

### 3.4. Hierarchical Linear Regression Models

After it was established that the assumptions of normality, linearity, multicollinearity, and homogeneity of variance were not violated, hierarchical linear regression analysis was applied, as shown in Table 4. There were no significant problems with multicollinearity because the variance inflation factor (VIF) for each control and predictor variable was less than 5. In the analyses of depression, anxiety, and stress, the Durbin–Watson coefficients were 2.14, 2.16, and 2.02, respectively. This demonstrated that the models did not have any significant autocorrelation problems [30].

#### 3.4.1. Depression

In the model with control variables, the value of the explained variance of the dependent variable depression was 8%; however, when control and predictor variables were taken together, the value increased to 37%, primarily because of resilience (β = −0.37, *p* < 0.01), hypomentalizing (β = 0.18, *p* < 0.05), and hypermentalizing (β = −0.16, *p* < 0.05). These findings showed that more resilience and hypermentalizing reduced depression and that more hypomentalizing increased depression. Socioeconomic status was a significant negative predictor of depression (β = −0.30, *p* < 0.01), which means that higher socioeconomic status implied less depression among healthcare workers.

#### 3.4.2. Anxiety

Resilience (β = −0.35, *p* < 0.01) and hypermentalizing (β = −0.17, *p* < 0.01), which were statistically significant negative predictors of anxiety, increased the value of the explained variance of the dependent variable anxiety from 10% in the model with control variables to 41% when control and predictor variables were combined. The findings suggest that as an aspect of an impaired mentalizing ability, hypomentalizing increased anxiety levels (β = 0.22, *p* < 0.01). When it came to the profession variable, medical technicians/nurses had a higher level of anxiety (β = 0.10, *p* < 0.05) than doctors. Furthermore, with the increase in socioeconomic status (β = −0.09, *p* < 0.05), anxiety decreased in the respondents.

#### 3.4.3. Stress

In the model with control variables, the value of the explained variance of the dependent variable stress was 6%, but when the control and predictor variables were combined, it increased to 43%. Resilience (β = −0.40, *p* < 0.01), hypomentalizing (β = 0.26, *p* < 0.01), and hypermentalizing (β = −0.15, *p* < 0.01) were all statistically significant predictors. The results indicated that higher levels of hypermentalizing and resilience implied lower levels of stress. The finding that hypermentalizing was an important negative predictor of stress indicated that a higher degree of confidence in one’s own ability to accurately assess intentional mental states reduced the degree of experiencing stress. The finding that hypomentalizing was an important positive predictor of stress indicated that the degree of uncertainty in one’s ability to accurately assess intentional mental states and the degree of stress increased together or decreased together. Along with the assessment of a higher socioeconomic status, the respondents also had less stress (β = −0.08, *p* < 0.05).

Age in the model with only control variables was a significant negative predictor of anxiety (β = −0.14, *p* < 0.01) and stress (β = −0.11, *p* < 0.05), which means that a higher number of years implied less anxiety and stress. Furthermore, in the model with only control variables, gender was a significant predictor of anxiety (β = 0.12, *p* < 0.05) and stress (β = 0.11, *p* < 0.05), which implied that women had higher levels of anxiety and stress. However, with the introduction of predictor variables into the models, age and gender ceased to be significant predictors of these two dimensions of mental health status. Marital status, number of children, and work environment were not statistically significant predictors of any of the three dimensions of mental health among healthcare workers.

## 4. Discussion 

This research aimed to investigate the role of resilience and mentalizing capacity in anticipating reported symptoms of depression, anxiety, and stress in healthcare workers during the COVID-19 outbreak. Although research that examined the relationship between mentalizing and mental health problems in a clinical sample is numerous, there is a noticeable lack of studies in the context of work psychology and occupational health. As far as we know, the relationship between these concepts has not been previously investigated on a sample of nurses and doctors.

Given that repeated exposure to stressors can result in a variety of mental health issues, the psychological effects of COVID-19 on healthcare workers who are working during the pandemic are a significant consideration to take into account [4]. This study showed that healthcare workers during the COVID-19 pandemic in Serbia not only had higher average values of depression, anxiety, and stress than the general population in Serbia during the lockdown [3] but also more than healthcare workers in China during the peak of the COVID-19 epidemic [31], as measured and scored in the same way.

The obtained results confirmed that resilience contributed to the explanation of each of the dimensions of mental health; more precisely, the greater the resilience of healthcare workers, the lesser the depression, anxiety, and stress they experienced, which is in line with the results of numerous studies conducted around the world during the pandemic [32]. Resilience is a dynamic process of adaptability to difficult life circumstances that includes several features of personal resources and was considered a protective factor against mental health problems in healthcare workers even before the outbreak of the COVID-19 pandemic [6]. It was demonstrated that during the COVID-19 pandemic, Serbian healthcare professionals’ resilience was inversely correlated with burnout, positively associated with subjective well-being, and attenuated the negative correlation between burnout and subjective well-being [6]. The research that was published as part of a large scientific project to which this study also belonged had great theoretical and practical significance because it shed light on the relationship between mentalizing, burnout syndrome, and resilience in healthcare workers during the COVID-19 pandemic [33]. According to the findings of that study, which was conducted on the same sample as the current study, resilience in healthcare workers enhances feelings of personal accomplishment at work and reduces emotional fatigue, while hypomentalizing in healthcare workers increases their emotional exhaustion and depersonalization, which, in turn, causes burnout [33]. Our findings are in line with research that showed resilience characteristics to be related to reduced levels of depressive symptoms and anxiety [34], and that resilience mediated the link between stress, anxiety symptoms, and depression during the outbreak of COVID-19 [35].

Deficits in mentalizing abilities are associated with higher prevalences of clinical levels of distress and greater emotion dysregulation [36,37]. Our findings showed that as an aspect of the impaired ability to mentalize, hypomentalizing, that is, difficulty effectively assessing the mental states that underlie behavior, increases the level of depression, anxiety, and stress among healthcare workers. Mentalizing is often simplistically understood as a synonym for the ability to empathize with other people, which is an especially important trait when it comes to professions that involve helping others. In fact, mentalizing includes a wide range of abilities that critically include the ability to see one’s own behavior as coherently organized by mental states and to be psychologically distinct from others [38]. Hypomentalizing or the tendency to infer less social meaning hinders mental processes and makes it difficult to understand how harmful some actions are to others, which leads to thinking about the relevance of the ability to mentalize in the work of healthcare providers. In contrast, a good ability to mentalize involves showing empathy, reflective listening, and genuine curiosity about revealing mental states during direct communication with the interlocutor. However, hypomentalizers have a tendency to ignore objective facts about the reasons for their behavior in communication with others and to judge mental states by guessing, which can lead them to wrong conclusions [39,40]. When it comes to impaired capacity for mentalizing in healthcare workers, it can reduce their ability to understand their own behavior and that of their patients or colleagues, which leads to interpersonal miscommunications, disputes, inconveniences, depersonalization, and job dissatisfaction [33], which, in turn, can result in burnout, depression, anxiety, and stress. All this is consistent with other research showing that good mentalizing ability is a safeguard for mental health [11,33,39,40]. Therefore, the lack of consideration of mental life phenomena that influence behavior by establishing assumptions and testing them in interpersonal interaction with colleagues and patients led to experiencing more distress among our respondents.

Mentalizing enables the ability to create positive and satisfying interpersonal relationships, gives meaning to one’s inner experience and the outer world, and promotes satisfying interpersonal functioning where a person manages to keep their sense of identity while feeling connected to others [41]. Scandurra et al. [42] showed that mentalizing, which is a mechanism by which individuals understand, modulate, and communicate their cognitive and emotional experiences, significantly protects against the onset of depressive and anxiety symptoms. This is in agreement with our findings, which showed that hypermentalizing, which in our respondents was understood as a higher degree of trust in one’s own ability to appropriately identify intentional mental states, reduced the levels of depression, anxiety, and stress. Similar findings were obtained by Lenzo et al. from a sample of 157 bereaved participants. In their study, it was shown that anxiety was negatively correlated with hypermentalizing, i.e., certainty about mental states, and in a positive correlation with hypomentalizing, i.e., uncertainty about mental states, which indicates a type of mentalizing impairment. Depression was also negatively correlated with hypermentalizing [43]. These findings suggest that hypermentalizing has a role in preventing depression, anxiety, and stress. The hypermentalizing scale that was used both in that and in our research refers mainly to the assessment of the degree of trust in the infallibility of one’s own assessment of one’s own and other people’s mental state [17,39]. In light of this, our research’s findings indicate that healthcare professionals who have a tendency to strongly believe in the accuracy of their assessments of their own and others’ mental states and who avoid verifying their assumptions about intentional mental states via direct communication with coworkers and patients, on the one hand, avoid frustrations and difficulties, which, in turn, reduces the experience of depression, anxiety, and stress. On the other hand, they continue to lack access to enough knowledge of their own and their coworkers’ and patients’ intentional mental states, which, as was previously established, is an aspect of hypermentalizing. In terms of sociodemographic, work, and COVID-19-related characteristics, our findings showed that age, in a model with only control variables, was a significant negative predictor of anxiety and stress, which means that older healthcare workers reported less anxiety and stress. This finding is in line with the results of research conducted by Biber et al. in the USA [44] and Yassin et al. in Jordan [45]. Moreover, in the model with only control variables, gender was a significant predictor of mental health status indicators of anxiety and stress in the sense that women had higher levels of anxiety and stress, which is in line with the studies conducted by Xiao et al. [26], Biber et al. [44], and Lai et al. [27]. However, with the introduction of predictors (resilience and mentalizing dimensions) into the models, age and gender ceased to be significant predictors of anxiety and stress.

Our findings also indicated the importance of good socioeconomic status in reducing depression, anxiety, and stress among healthcare workers, which is in line with the results of earlier research suggesting that people with lower socioeconomic status are more prone to mental health problems [46,47]. Profession was a significant predictor only in the regression model of anxiety. Nurses had higher levels of anxiety than doctors, which is consistent with previous findings from China [27].

### 4.1. Practical Implications

Distress during the COVID-19 pandemic is a reaction to difficult circumstances that surround all people, especially healthcare workers. Mentalizing abilities offer increased flexibility in emotional understanding, thereby helping to reduce some of the most severe effects of stress. Increasing mentalizing may serve to increase the capacity to bear the negative consequences of potentially traumatic experiences [48]. Findings from the literature suggest that resilience training can be beneficial for healthcare professionals, as resilience is considered the ability to adapt to adversity, maintain balance, maintain control, and cope with external stressors [5]. Because resilience and mental health are closely related and depend on the interaction between personal and broader social factors, such as safety and accessibility to education and employment, effective strategies to support mental health and promote resilience that focus on self-efficacy and community participation are especially important after crisis situations [49], such as a pandemic. The gold standard of care should be the development of online interventions centered on resilience and the availability of psychological support for easier stress management during the COVID-19 pandemic and dealing with the long-term consequences related to one’s quality of life, personal functioning, and overall well-being [50].

Although our findings have relevant implications for clinical practice, as they emphasize the importance of mentalizing and resilience for the prevention of mental disorders in healthcare workers in the context of a chronic pandemic and increased social stressors, it should be noted that additional research is necessary to examine in more detail the factors related to COVID-19 that have an impact on the mental health of healthcare workers.

### 4.2. Limitations

The current study has certain limitations. The data were cross-sectional, and thus, causation cannot be sufficiently established and reverse causation cannot be ruled out. Another limitation that must be taken into account is that healthcare workers from only one tertiary care hospital were included, and thus, the results cannot be generalized to the entire workforce of healthcare professionals. Additionally, response biases, which can often be difficult to eliminate in self-reported survey research like this one, may have influenced respondents’ opinions, thus limiting the results of this study.

## 5. Conclusions

In summary, the most important conclusions of the current study were that resilience and hypermentalizing in healthcare workers reduced depression, anxiety, and stress, and that hypomentalizing, as a failure in mentalizing characterized by low certainty about the mental state of oneself and others, increased their depression, anxiety, and stress during the COVID-19 pandemic.

Since resilience and the mentalizing capacity were shown to play very important roles when it came to preventing mental health problems, there is an urgent need to establish and implement strategies to foster resilience and enhance the capacity for mentalizing among healthcare workers in order to reduce the devastating impact of the COVID-19 pandemic on mental health.

## Figures and Tables

**Table 1:** Characteristics of the study participants.

Characteristics		Sample Size (*N* = 406)
Age (years), mean ± SD		40.11 ± 9.41
Gender, *N* (%)	Male	139 (34.2)
	Female	267(65.8)
Profession, *N* (%)	Doctors	141 (34.7)
	Nurses	265 (65.3)
Work environment, *N* (%)	COVID-19 frontline healthcare workers	203 (50)
	Non-frontline healthcare workers	203 (50)
Marital status, *N* (%)	Married	291 (71.7)
	Single	115 (28.3)
Number of children, *N* (%)	No children	120 (29.6)
	One child	111 (27.3)
	Two or more children	175 (43.1)
Socioeconomic status, *N* (%)	Very poor	12 (3)
	Poor	71 (17.5)
	Good	229 (56.4)
	Very good	78 (19.2)
	Excellent	16 (3.9)

**Table 2:** Descriptive statistics of used measures.

Scale	Min.	Max.	Mean	SD	Skew	Kurt	*α*
Depression	0	42	10.63	10.63	1.04	0.26	0.96
Anxiety	0	42	10.19	9.44	0.99	0.32	0.93
Stress	0	42	17.87	11.98	0.26	−0.92	0.96
Resilience (BRS)	1.00	5.00	3.21	0.74	−0.08	0.00	0.76
Hypermentalizing (RFQ-c)	0.00	3.00	1.19	0.88	0.35	−0.96	0.82
Hypomentalizing (RFQ-u)	0.00	2.50	0.57	0.62	1.21	0.68	0.70

**Table 3:** Correlations between the investigated variables.

	Depression	Anxiety	Stress	BRS	RFQ-c	RFQ-u	Gender	Age	M.S.	N.C.	SE.S.	Profession
Anxiety	0.68 **											
Stress	0.69 **	0.72 **										
BRS	−0.51 **	−0.51 **	−0.53 **									
RFQ-c	−0.42 **	−0.43 **	−0.49 **	0.36 **								
RFQ-u	0.40 **	0.46 **	0.53 **	−0.27 **	−0.61 **							
Gender	0.13 **	0.25 **	0.17 **	−0.18 **	−0.11 *	0.13 **						
Age	−0.04	−0.09	−0.14 **	0.07	0.18 **	−0.15 **	0.02					
M.S.	−0.02	−0.07	0.02	0.08	0.01	0.05	−0.06	−0.18 **				
N.C.	−0.04	0.09	−0.11 *	−0.06	0.11 *	−0.06	0.12 **	0.46 **	−0.48 **			
SE.S.	−0.28 **	−0.20 **	−0.19 **	0.19 **	0.07	−0.13 **	−0.13 **	−0.10 *	0.01	−0.05		
Profession	0.10 *	0.23 **	0.10 *	−0.09	−0.06	0.12 *	0.47 **	−0.04	−0.07	0.17 **	−0.10 *	
W.E.	−0.10 *	−0.10 *	−0.13 **	0.08	0.13 **	−0.11 *	−0.15 **	0.08	−0.02	0.07	0.15 **	−0.06

Note: ** *p* < 0.01, * *p* < 0.05, BRS—brief resilience scale, RFQ-c—reflective function questionnaire certain, RFQ-u—reflective function questionnaire uncertain, M.S.—marital status, N.C.—number of children, SE.S.—socioeconomic status, W.E.—work environment (COVID-19 frontline or non-frontline).

**Table 4:** Hierarchical linear regression analysis of the relationship among DASS-42 dimensions, resilience, and mentalizing.

	Outcome Variable: Depression					
	Control Variables			Control Variables and Predictors		
	*β*	*t*	VIF	*β*	*t*	VIF
Gender	0.07	1.41	1.33	−0.11	−0.24	1.36
Age	−0.04	−0.73	1.33	0.06	1.45	1.37
M.S.	−0.05	−1.01	1.31	−0.02	−0.55	1.33
N.C.	−0.07	−1.28	1.69	−0.09	−1.81	1.71
Profession	0.04	0.83	1.34	0.04	0.97	1.35
SE.S.	−0.27 **	−5.57	1.05	−0.17 **	−4.19	1.10
W.E.	−0.03	−0.71	1.06	0.00	−0.00	1.06
BRS				−0.37 **	−8.60	1.23
RFQ-c				−0.16 **	−3.12	1.78
RFQ-u				0.18 **	3.56	1.67
R²	**0.10**			**0.39**		
adj. R²	**0.08**			**0.37**		
F Ch.	6.50 **			61.96 **		
	**Outcome Variable: Anxiety**					
	**Control Variables**			**Control Variables and Predictors**		
	***β***	**t**	**VIF**	***β***	**t**	**VIF**
Gender	0.12 *	2.36	1.33	0.03	0.85	1.36
Age	−0.14 **	−2.74	1.33	−0.03	−0.81	1.37
M.S.	−0.01	−0.23	1.31	0.01	0.34	1.33
N.C.	0.06	1.11	1.69	0.05	1.13	1.71
Profession	0.10 *	1.97	1.34	0.10 *	2.30	1.35
SE.S.	−0.19 **	−4.10	1.05	−0.09 *	−2.42	1.10
W.E.	−0.03	−0.67	1.06	0.00	0.13	1.06
BRS				−0.35 **	−8.40	1.23
RFQ-c				−0.17 **	−3.41	1.78
RFQ-u				0.22 **	4.59	1.67
R²	**0.11**			**0.42**		
adj. R²	**0.10**			**0.41**		
F Ch.	7.43 **			71.31 **		
	**Outcome Variable: Stress**					
	**Control Variables**			**Control Variables and Predictors**		
	***β***	**t**	**VIF**	***β***	**t**	**VIF**
Gender	0.11 *	2.08	1.33	0.17	0.38	1.36
Age	−0.11 *	−2.05	1.33	0.00	0.18	1.37
M.S.	−0.03	−0.58	1.31	−0.00	−0.10	1.33
N.C.	−0.07	−1.24	1.69	−0.09	−1.91	1.71
Profession	0.00	0.04	1.34	−0.00	−0.07	1.35
SE.S.	−0.19 **	−3.94	1.05	−0.08 *	−2.07	1.10
W.E.	−0.05	−1.03	1.06	−0.01	−0.29	1.06
BRS				−0.40 **	−9.66	1.23
RFQ-c				−0.15 **	−3.15	1.78
RFQ-u				0.26 **	5.44	1.67
R²	**0.08**			**0.45**		
adj. R²	**0.06**			**0.43**		
F Ch.	5.12 **			88.05 **		

Note: ** *p* < 0.01, * *p* < 0.05. F Ch.—change in F, adj. R²—adjusted R-squared, BRS—brief resilience scale, RFQ-c—reflective function questionnaire certain, RFQ-u—reflective function questionnaire uncertain, M.S.—marital status, N.C.—number of children, SE.S.—socioeconomic status, W.E.—work environment (COVID-19 frontline or non-frontline); statistically significant correlations are bolded.

## Data Availability

The data presented in this study are available on request from the corresponding author.
